# Arthroscopic approaches to and anatomy of the shoulder joint of cattle: a cadaver study

**DOI:** 10.1186/s12917-020-02337-z

**Published:** 2020-05-24

**Authors:** Mahmoud Fadul, Alois von Rotz, Maher Alsaaod, Reiichiro Sato, Adrian Steiner

**Affiliations:** 1 Clinic for Ruminants, Department of Clinical Veterinary Medicine, Vetsuisse-Faculty, University of Berne, CH-3001 Bern, Switzerland; 2 Department of Surgery and Anesthesia, Faculty of Veterinary Medicine, University of Khartoum, P.O. Box 32, Khartoum North, Khartoum, Sudan; 3 Division of Veterinary Anatomy, Vetsuisse-Faculty, University of Berne, CH-3001 Bern, Switzerland; 4 School of Veterinary Medicine, Azabu University, Sagamihara, Kanagawa 252-5201 Japan

**Keywords:** Arthroscopy, Anatomy, Cattle, Shoulder joint, Lameness

## Abstract

**Background:**

Arthroscopic surgery is described as a minimally invasive technique for diagnosis, exploration and treatment of joint disorders. It allows intraarticular structures to be assessed accurately, thereby improving the diagnostic capabilities, and it broadens the spectrum of surgical techniques feasible for treatment of articular pathologies in cattle.

This study aimed to assess for cattle the described arthroscopic approaches to the shoulder joint of horses, and to describe the appearance of the corresponding intraarticular structures of the shoulder joint. Additionally, to perform histological examination where tissues were identified and assessed arthroscopically, but the tissue type was uncertain using cadaveric limbs from cattle of different age categories without any signs of orthopedic diseases of the front limbs.

**Results:**

An anatomic and arthroscopic investigation with 34-cadaveric forelimbs from 20-cattle was performed. The arthroscope was inserted either immediately cranial or 1-cm caudal to the tendon of the infraspinatus muscle for the cranial and caudal approaches, respectively. The shoulder joints were examined with the limbs in either horizontal non-pulled position, abducted non-pulled position using a three-pod limb holder adjustable in height, or horizontal manually pulled position. Arthroscopy was performed using a rigid 30°arthroscope (18-cm length, 4-mm outer diameter) to view the synovial pouches with their synovial villi and the following structures: cranial rim of the glenoid, cranial portion of the humeral head, incisura-glenoidalis, caudal rim of the glenoid, caudal portion of the humeral head, and cranial and caudal cul-de-sac. Abduction of the limb allowed improved visualization of the lateral portion of the joint. Pulling the limb facilitated investigation of the medial portion of the joint. Generally, the distention range was higher in younger as compared to adult cattle, and visualization of the medial portion of the joint was, therefore, facilitated in younger animals. The main complications observed were subcutaneous fluid extravasations and partial-thickness articular cartilages wear-lines.

**Conclusion:**

The described arthroscopic techniques allowed good overall visualization of the most relevant anatomical structures within the healthy cadaveric joint. Further investigations are warranted to evaluate the diagnostic and therapeutic applications of these techniques and the prognosis of arthroscopic surgery as a tool for the treatment of joint lesions.

## Background

The shoulder joint is a spherical joint, consisting of the scapular glenoid cavity and the humeral head surrounded by a group of tendons that support the joint and function as ligaments. These tendons are the biceps brachii tendon cranially, the supraspinatus and infraspinatus laterally, the teres minor and deltoideus caudally and the subscapularis medially. In cattle, additional ligamentous structures do not exist around the shoulder joint [1–3].

In cattle, shoulder lameness is infrequent, and few reports are available. Disorders related to the shoulder region such as septic arthritis, bursitis of the bicipital bursa and infection of the sub-tendinous bursa of the infraspinatus muscle affect individual animals and usually appear unilaterally [4–9]. Localized swelling, pain at palpation, muscle atrophy, swinging-limb lameness and abduction of the affected limb while standing and walking were found as typical clinical features of shoulder lesions. Distinguishing between various anatomical structures of the shoulder region through clinical palpation is challenging [9]. It is difficult to confirm the diagnosis and obtain radiographs, due to the complex anatomy of the region [6, 7, 10].

Generally, diagnosis of joint disorders relies on clinical, radiographic and ultrasonographic examination followed by arthrocentesis for macroscopic, cytological and microbiological analysis of synovial fluid [11–13]. In cattle, various surgical interventions have been described for the treatment of arthritis and other joint disorders. They include joint lavage, arthrotomy, joint resection and arthrodesis [14–17]. Arthroscopic surgery has been described as a minimally invasive technique for diagnosis, exploration and treatment of joint disorders. It allows intraarticular structures to be assessed accurately, thereby improving the diagnostic capabilities, and it broadens the spectrum of surgical techniques feasible for treatment of articular pathologies in cattle [12, 14, 18].

However, arthroscopy is uncommon in cattle practice due to the high instrument costs, the cattle practitioners’ lack of experience and requirement for general anesthesia [18, 19]. Therefore, show cows, breeding bulls and high genetic value cows are more likely to be selected for arthroscopic surgery [20]. In cattle, arthroscopic surgery was first reported for the treatment of osteochondritis dissecans of the stifle joint [21]. Chronic septic arthritis of the tibiotarsal, antebrachiocarpal and fetlock joints was successfully treated in 12 out of 14 cattle (86%), using arthroscopic lavage followed by intraarticular gentamicin-impregnated collagen sponge implantation [12, 14]. Furthermore, cases of osteomyelitis and osteochondrosis recovered after arthroscopic surgery [22, 23].

Recently, several studies described arthroscopic approaches to the joints of cattle including the relevant arthroscopic anatomy of the fetlock, carpus, tarsus and stifle [24–29]. Description of arthroscopic approaches and anatomy of the shoulder joint is available for horses but not for cattle [30].

### Aims

This study firstly aimed to assess for cattle the described arthroscopic approaches to the shoulder joint of horses; and secondly, to describe the appearance of the corresponding intraarticular structures of the shoulder joint using cadaveric limbs from cattle of different age categories without any signs of orthopedic diseases of the front limbs. In addition, to perform histological examination where tissues were identified and assessed arthroscopically, but the tissue type was uncertain.

## Results

### Anatomy

The anatomical study was conducted prior to the arthroscopic study to examine whether the respective puncturing sites described for the horse were feasible for cattle too without causing any unnecessary tissue damage and to optimize the identification process of the various anatomical structures during arthroscopy. The anatomical study produced the same results as have been described in the literature [1–3]. Gross dissection of the shoulder joint demonstrated the palpable structures. The scapular spine, tendon of infraspinatus muscle and the major tubercle of the humerus were used as landmarks for the arthroscopic entry procedures.

### Arthroscopy

Arthroscopic examination of the shoulder joint was successfully performed through the cranial and caudal approaches in all cases. At the beginning of the study, the arthroscopic examination took approximately 45 min for each approach, and the duration of the examination decreased to a total of 20 min by improving the proficiency of the surgeon.

#### Horizontal non-pulled position

**Fig. 1:**
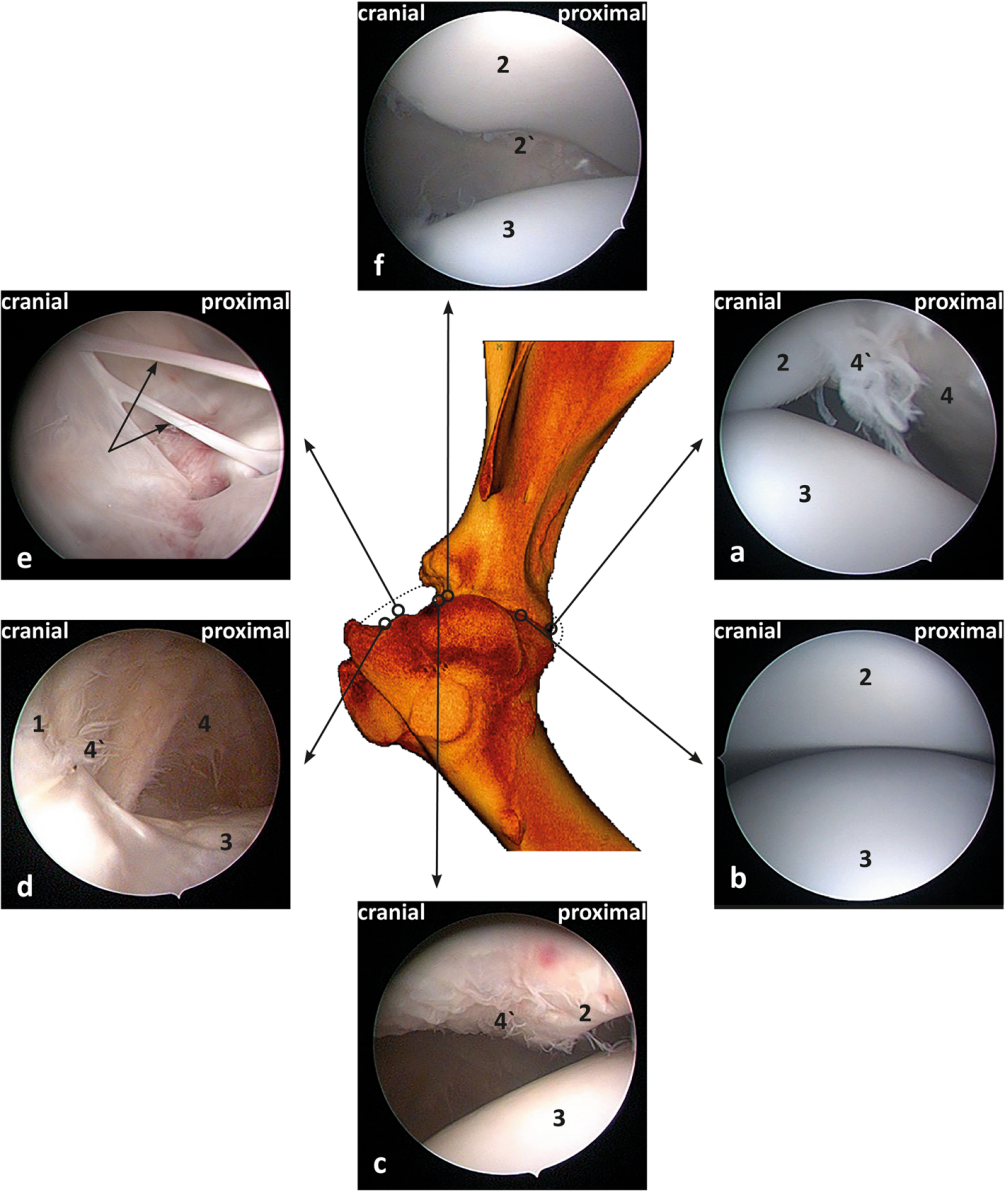
Arthroscopic views of the left shoulder joint within the limb in horizontal non-pulled position (**a**-**f**). **a** Caudal aspect of the joint; **b** middle aspect of the joint lateral view; **c**, **d**, **e** and **f** cranial aspect of the joint. (1) Cranial cul-de-sac; (2) glenoid rim; (2`) incisura glenoidalis; (3) humeral head; (4) synovial membrane; (4`) synovial villi. Dotted arrows (E) refer to the tissue strands including arterioles and venules


With the limb in a horizontal non-pulled position, the systematic examination of the joint for the cranial approach began by passing the arthroscope beneath the cranial border of the infraspinatus tendon with the tip of the arthroscope pointed to the caudal aspect of the joint “See Additional file 1”. Orientation within the joint was established by identifying the rim of the glenoid dorsally, the humeral head ventrally and the synovial membranes of the caudal cul-de-sac caudally and medially. Apart from the synovial villi located around the glenoid rim dorsally (Fig. 1a), several synovial villi were also observed in the caudal cul-de-sac. Afterwards, withdrawing the arthroscope slowly along the lateral aspect of the joint allowed the examination of the lateral rim of the glenoid dorsally and the humeral head ventrally (Fig. 1b).

**Additional file 1** Arthroscopic approaches to and anatomy of the shoulder joint of cattle: A cadaver study; cranial approach. A movie shows the arthroscopic examination of a cadaveric shoulder joint through the cranial approach with the limb in a horizontal non-pulled position.


**Fig. 2:**
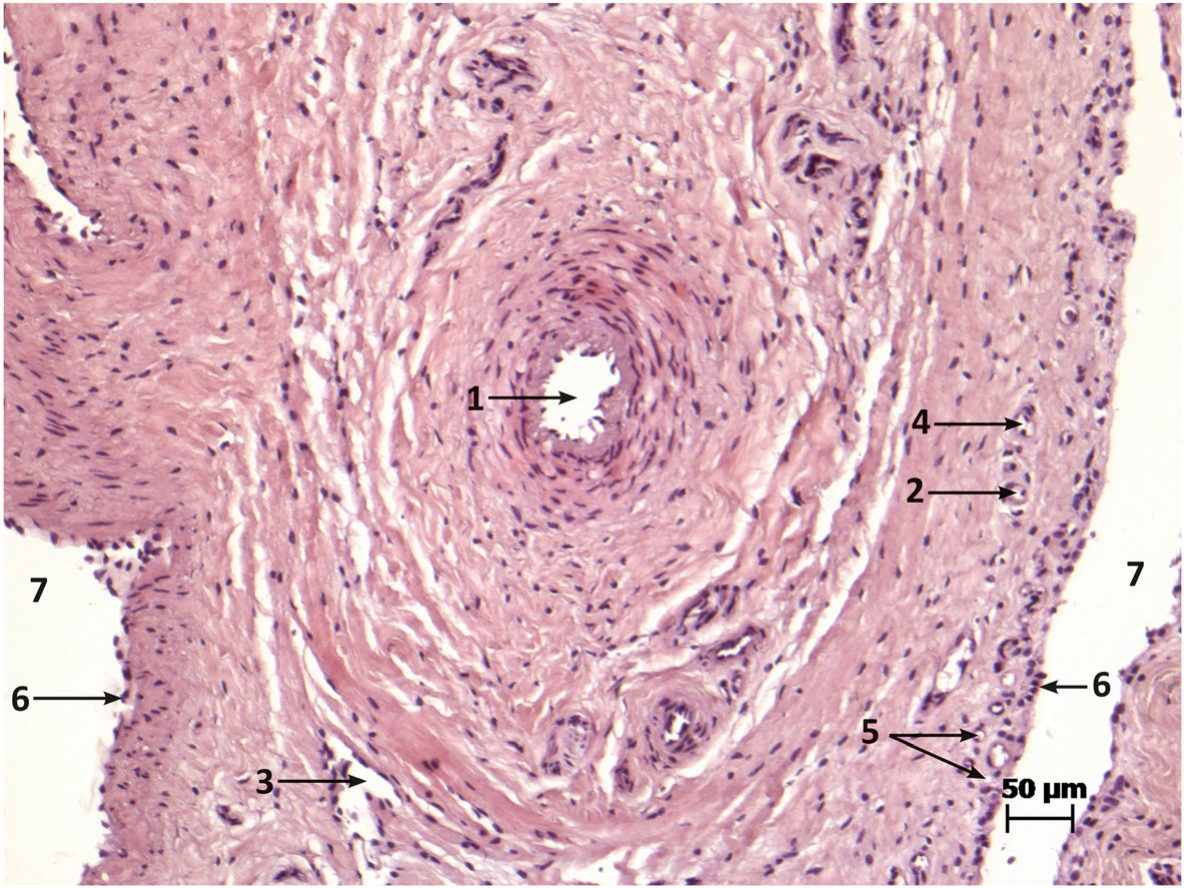
Histological findings of one of the stranded bands; (1) Artery (2) arterioles (3) vein (4) venule (5) blood capillaries (6) synovial layer (7) synovial space

At half the distance between the cranial and caudal cul-de-sac, the arthroscope was carefully inserted below the glenoid and over the humeral head towards the medial side of the joint; the articular surface of both the glenoid dorsally and the humeral head ventrally were examined by rotating the arthroscope (360°). The synovial membrane medially was also investigated and found to be smooth and glistening, and the medial synovial villi were partially visible. Then, the arthroscope was withdrawn to the lateral aspect of the joint and pushed cranially, where the lateral aspects of the cranial glenoid rim medially and dorsally and the humeral head ventrally were visible including the synovial villi attached dorsally to the glenoid rim (Fig. 1c). Thereafter, the cranial cul-de-sac of the joint was examined from lateral to medial, and complete rotation of the arthroscope (360°) around its long axis allowed the cul-de-sac to be examined dorsally and ventrally in further detail (Fig. 1d). In addition, thin tissue strands stretching between the cranial cul-de-sac and small fossae in the cranial portion of the humeral head ventrally in both young and adult animals were observed in most shoulders (26/30) (Fig. 1e). Histological examination of one of the stranded bands revealed the presence of large and small blood vessels (arterioles and venules) (Fig. 2). Then, the arthroscope was directed medially, and the craniomedial portions of the glenoid rim dorsally, the craniomedial, of the humeral head ventrally and the synovial membranes medially were investigated. Thereafter, the incisura glenoidalis was examined dorsomedially by passing the arthroscope slightly in a caudal direction at the craniomedial aspect of the joint (Fig. 1f).

As an alternative approach, inserting the arthroscope 1-cm caudal to the tendon of the infraspinatus muscle allowed the investigation of the same structures as mentioned above, but they were visualized in the opposite order “See Additional file 2”. Visualization of the medial aspects of the caudal cul-de-sac was restricted to a minimum through the cranial approach and full visualization of the medial aspects of the cranial cul-de-sac was impeded through the caudal approach. In addition, manipulation of the arthroscope through the caudal approach was more difficult as compared to the cranial approach, and this was mainly observed in adults rather than calves.


**Additional file 2** Arthroscopic approaches to and anatomy of the shoulder joint of cattle: A cadaver study; caudal approach. A movie shows the arthroscopic examination of a cadaveric shoulder joint through the caudal approach with the limb in a horizontal non-pulled position.


Unimpeded and complete visualization of the mentioned intra-articular structures of the shoulder joint was achieved in all limbs examined except for the incisura glenoidalis which was visualised in 29/30 and the medial portion of the glenoid rim in 24/30 limbs.

#### Abducted non-pulled position and horizontally pulled limb

**Fig. 3:**
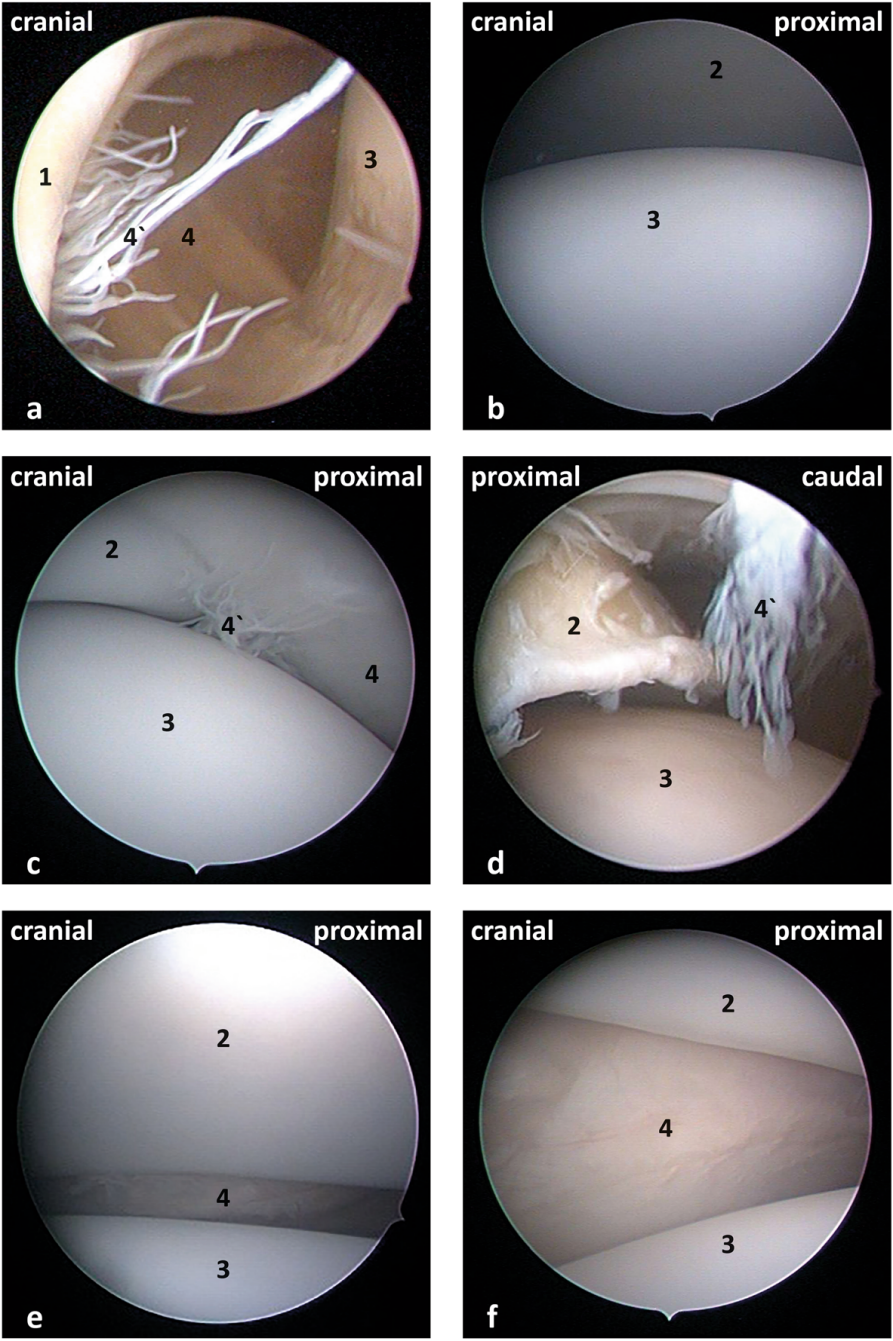
Arthroscopic views of the left shoulder joint with the limb in abducted non-pulled position (**a**-**d**) and horizontal pulled position (**e** and **f**) for the cranial approach. **a** Cranial aspect of joint; **b**, **e** and **f** middle aspect of the joint; (**c** and **d**) caudal aspect of the joint; (1) Cranial cul-de-sac; (2) glenoid rim; (3) humeral head; (4) synovial membrane; (4`) synovial villi and (5) caudal cul-de-sac

With the limb in an abducted non-pulled position, visualization of the lateral portion of the humeral head ventrally and the lateral border of the glenoid rim dorsally was greatly facilitated during both the cranial and caudal approaches. The cranial cul-de-sac, synovial villi, cranial portion of the humeral head, caudal rim of the glenoid, caudal portion of the humeral head and synovial villi were investigated (Fig. 3a, b, c and d). Pulling the limb horizontally facilitated entering the joint space, allowing for an improved visibility of the medial portions of the glenoid rim dorsally, the humeral head ventrally and the synovial membrane covered with a brush of short synovial villi medially (Fig. 3e). Investigating the medial aspect of the joint was easier in young animals (Fig. 3f) compared to adults (Fig. 3e). Particularly, the previously mentioned structures were nicely visualised in all the 10 shoulder joints that underwent arthroscopy in the abducted non-pulled and the horizontally pulled position.

Two intraoperative complications occurred during arthroscopy in all cadavers: (i) extravasations of irrigation fluid subcutaneously which were more prominent in cadavers of calves as compared to adults and (ii) partial-thickness articular cartilage wear lines due to the insertion, reinsertion and manipulation of the arthroscope.

## Discussion

This study assessed for cattle the described arthroscopic approaches to the shoulder joint of horses and described normal arthroscopic findings of two approaches to the shoulder joint in sound cattle. Arthroscopic visualization of the shoulder joint was performed systematically through skin incisions located either cranial or caudal to the tendon of the infraspinatus muscle.

In the present study, the described anatomical landmarks and arthroscopic approaches that have been used to insert the arthroscope and to assess the shoulder joint in cattle were similar to the landmarks and approaches that had already been described for horses [30–32]. The anatomical variation between cattle and horses concerned the scapular spine which was more often visible and always palpable in cattle as compared to horses [1].

Although arthroscopic approaches to and normal arthroscopic anatomy of many joints in cattle have been described, description of the arthroscopic approaches to and normal arthroscopic anatomy of the shoulder joint in cattle was not available [24–29]. To our knowledge, this is the first description of the arthroscopic approaches to and the intraarticular arthroscopic anatomy of the shoulder joint in cattle. In their review, Nichols and Lardé, suggested inserting the arthroscope either cranial or caudal to the tendon of the infraspinatus muscle in cattle as described in horses and as advocated in the current study [18].

The shoulder area anatomy as well as the considerable distance of the joint capsule from the skin surface, as well as the considerable distance of the joint capsule from the skin surface, complicate the clinical diagnosis of shoulder joint diseases and injuries through palpation, radiography, arthrocentesis and arthroscopic examination. For the shoulder joint; the arthroscopic investigation in horses, and arthrocentesis in cattle were found to be much more difficult compared to other joints [6, 7, 9, 30, 31]. In cattle, recent studies classified the shoulder joint as the second most difficult joint for entering and evaluation following the hip joint [19, 33]. In adult cattle, the surgeon has to apply more pressure to the arthroscope for manipulation through the caudal approach, and this may lead to arthroscope damage, while the cranial approach gave similar or better visibility with lower damage risk to the arthroscope. One explanation for this difficulty might be the distance from the skin to the joint capsule is longer in the caudal as compared to the cranial approach. This finding is in agreement with previous findings described in horses [30, 31].

In the current study, evaluation of the cranial, lateral and caudal portions of the shoulder joint via both the cranial and caudal approaches with the limbs in horizontal and non-pulled position was possible. A number of structures were efficiently evaluated: the cranial cul-de-sac, the cranial rim of the glenoid, cranial aspect of the humeral head, incisura glenoidalis, lateral rim of the glenoid, latero-proximal aspect of the humeral head, the caudal rim of the glenoid, caudal aspect of the humeral head and caudal cul-de-sac. Exploration of the full extent of the medial aspect of the joint was not possible neither in calves nor adults. However, certain parts of the medial glenoid rim, medial synovial membrane and proximal medial portion of the humeral head were more easily accessible in calves rather than adults. Pulling the limb manually in a horizontal direction facilitated the manipulation of the arthroscope to examine the medial joint aspects. Manipulation of the arthroscope in cadaveric limbs as compared to cattle under general anesthesia might be more difficult due to the loss of elasticity of the joint capsule [18]. The abduction of the limb allowed to improve visualization of the lateral portion of the humeral head as has already been described in horses [30–32]. It might be concluded that one arthroscopic portal is sufficient to examine the shoulder joint; however, it was beneficial to either extend or abduct the joint during the examination in order to improve visibility of certain structures.

The selection of the approach of the arthroscope in clinical cases may depend on the location of the lesion. From the current study, we propose using the cranial arthroscopic approach for lesions of the caudal portion and the portal 1-cm caudal to the palpable border of the tendon of the infraspinatus muscle to allow for the instrument access. The caudal insertion of the arthroscope might be ideal for accessing lesions of the cranial area of the joint, while the cranial portal is used for the instruments. Nichols and Anderson, proposed that in cattle with joint disease, the ideal arthroscopic and instrument portals may not necessarily to be identical to those suggested in healthy cattle [18]. However, in the shoulder joint, we cannot imagine any other practically applicable approach for arthroscopic evaluation.

Nutrient foramina are important since they provide a pathway for the entrance of nutrient arterioles and venules into the bone. The histological examination of the observed stretched strands located at the cranial portion of the joint revealed the presence of arterioles and venules, allowing for the supply of blood to the humerus. Considering the number and location of these structures, intra-operative laceration should be avoided [34]. In a study by Mansur et al., 2% of 253 examined human humeral heads did not show any nutrient foramina [34]. This finding is similar to that experienced in the cases of the current study in shoulder joints of cattle.

Various complications may occur during arthroscopic surgeries. In the present study, extravasations of the irrigation fluid subcutaneously occurred to some degree in all the examined shoulders, but it was more obvious in the calves’ cadavers. The reason for fewer extravasations in adults compared to calves might be that the skin is less tight in calves as compared to adults. In horses, this complication was also reported in shoulder arthroscopy [30]. The extended time of the surgeries, besides puncturing the joint capsule at more than one site and applying high perfusion pressure were the main reasons for the fluid extravasations to occur [30]. In the current study, partial-thickness articular cartilage wear lines were observed in all shoulders examined. Although these lesions occurred already during the first insertion of the instruments into the joint, reinsertion and manipulating the arthroscope within the joint added additional lesions. Partial-thickness articular cartilage wear lines were also described in cattle carpus and stifle arthroscopy [26, 27, 29]. As mentioned before, puncturing and manipulating of the arthroscope within the shoulder joint is demanding [19, 33], this is most likely the explanation for the occurrence of the partial-thickness articular cartilage wear lines in our study. The described complications can be avoided or its occurrence at least be reduced by applying a minimal perfusion pressure, gently inserting and manipulating the arthroscope and avoiding to perform the intervention through more than one optical portal.

## Conclusion

Arthroscopy of the shoulder joint in cattle is a minimally invasive surgical technique that allows a good overall view of most of the relevant intraarticular structures, except for some aspects of the medial parts of the joint. The cranial cul-de-sac is better visualized through the cranial approach and the caudal cul-de-sac through the caudal approach. Extending the limb in horizontal direction improves visualization of the joint space and manipulation of the arthroscope between the cartilaginous surfaces, while abducting the limb improved visualization of the lateral aspects of the joint. Further clinical investigations are necessary to evaluate the diagnostic, therapeutic and prognostic properties of arthroscopy and surgical interventions under arthroscopic control in the shoulder joint of cattle in the presence of joint lesions.

## Methods

### Animals

Thirty-four cadaveric forelimbs (19 right and 15 left limbs) of 20 cattle with a history of no orthopedic disorder of the front limbs were included in the study. Seven limbs originated from 4 adults with an age of 3.7 ± 1.7 years (Mean ± SD), a bodyweight of 671.3 ± 71.8 kg and 27 limbs from 14 young animals with an age of 34.4 ± 50.6 days and a bodyweight of 70.7 ± 41.7 kg. Breeds included Holstein Friesian (*n* = 8), Red Holstein (*n* = 5), Swiss Fleckvieh (*n* = 4), Swiss Braunvieh (*n* = 2) and Rhätisches Grauvieh (*n* = 1). The study was divided into 2 parts: for the anatomical study, four cadaveric forelimbs and for the arthroscopic investigation, 30 forelimbs were used.

### Anatomical study

The anatomical study was conducted prior to the arthroscopic study to determine the ideal site of entering the joint and to improve the identification process of the various anatomical structures during the arthroscopy. Three randomly selected shoulder joints originated from two healthy calves with an age of 12 and 30 days and a bodyweight of 52 and 70 kg, respectively and that did not show any signs of orthopedic disorder of the front limbs were injected with blue-colored silicon (ELASTOSIL® RT K). Two G18 (1.2 × 40 mm) hypodermic needles were inserted cranial and caudal to the infraspinatus tendon: one to inject the silicon into the joint cavity and the second one to allow the synovial fluid to drain. A total volume of 20–30 ml of the silicon compound was injected until its outflow from the second needle was evident. To ensure good diffusion of the silicon within the joint, the joint was flexed and extended through a normal range of motion several times after the injection. Then, the limbs were placed in cold water at 10–15 °C for 1 day after which a topographical dissection was made to reveal the anatomical structures. Moreover, a fourth foreleg from an adult cow cadaver with an age of 5.2 years and a bodyweight of 675 kg was frozen in − 20 °C for 48- h and sliced sagittally using an electric band saw; each section was approximately 15 mm thick, and tap water was used to rinse the sections. Photographs of each injected limb and of the sections were taken (Panasonic, Lumix, DMC-FZ50) to allow comparisons with subsequent arthroscopic findings.

### Instruments for arthroscopy

For the arthroscopic investigation, a rigid 30° arthroscope with a length of 18-cm, an outer diameter of 4-mm and a sleeve outer diameter of 5.5 mm with two connections **(**STORZ Endoskop Produktions GmbH, Tuttlingen, Germany**)** was used. An Endovision Veterinary Video Camera (Telecam SL II; STORZ Endoskop Produktions GmbH, Tuttlingen, Germany) transmitted the endoscopic pictures to a colour screen. The use of the Karl Storz AIDA WD 250® clinical documentation system (Advanced Image and Data Archiving System; STORZ Endoskop Produktions GmbH, Tuttlingen, Germany) allowed the continuous digital recording of the endoscopic data for further analysis later on. An irrigation system (Exacta-Vet®, Schoch Electronics AG, and Switzerland) was used to maintain joint distention and to provide continuous joint pressure during the procedure. Tap water was used as irrigation fluid.

### Arthroscopic approach

Thirty cadaveric right (*n* = 17) and left (*n* = 13) forelimbs from 18 cattle within 24-h after euthanasia were used for the arthroscopic study; 6 limbs belonged to 4 adults (age of 3.7 ± 1.7 years (Mean ± SD), a bodyweight of 671 ± 71.8 and 24 limbs to 14 young stock (age of 36.3 ± 52.8 days, a bodyweight of 72.2 ± 44.7). A hypodermic needle was placed immediately cranial to the tendon of the infraspinatus muscle into the joint and 20–60 ml of tap water was injected (depending on the cadavers’ weight) to distend the joint capsule. Then, the needle was removed, leaving the joint distended.

For the cranial approach, a vertical 5-mm stab incision with a #11 scalpel blade was made at the site of the needle insertion and proximal to the notch dividing the major tubercle of the humerus into cranial and caudal components. The arthroscopic sleeve with the blunt trocar was introduced into the joint in a slightly distal and caudal direction, performing gentle rotating movements. For the caudal approach, a vertical stab incision was made 1-cm caudal to the palpable border of the infraspinatus tendon and 1-cm proximal to the caudal component of the major tubercle of the humerus, and the arthroscopic sleeve with the blunt trocar was introduced into the joint in a slightly proximal and cranial direction, performing gentle rotating movements. At removal of the trocar, the correct placement of the instrument was immediately evident by the passive outflow of the injected tap water from the sleeve.

**Fig. 4:**
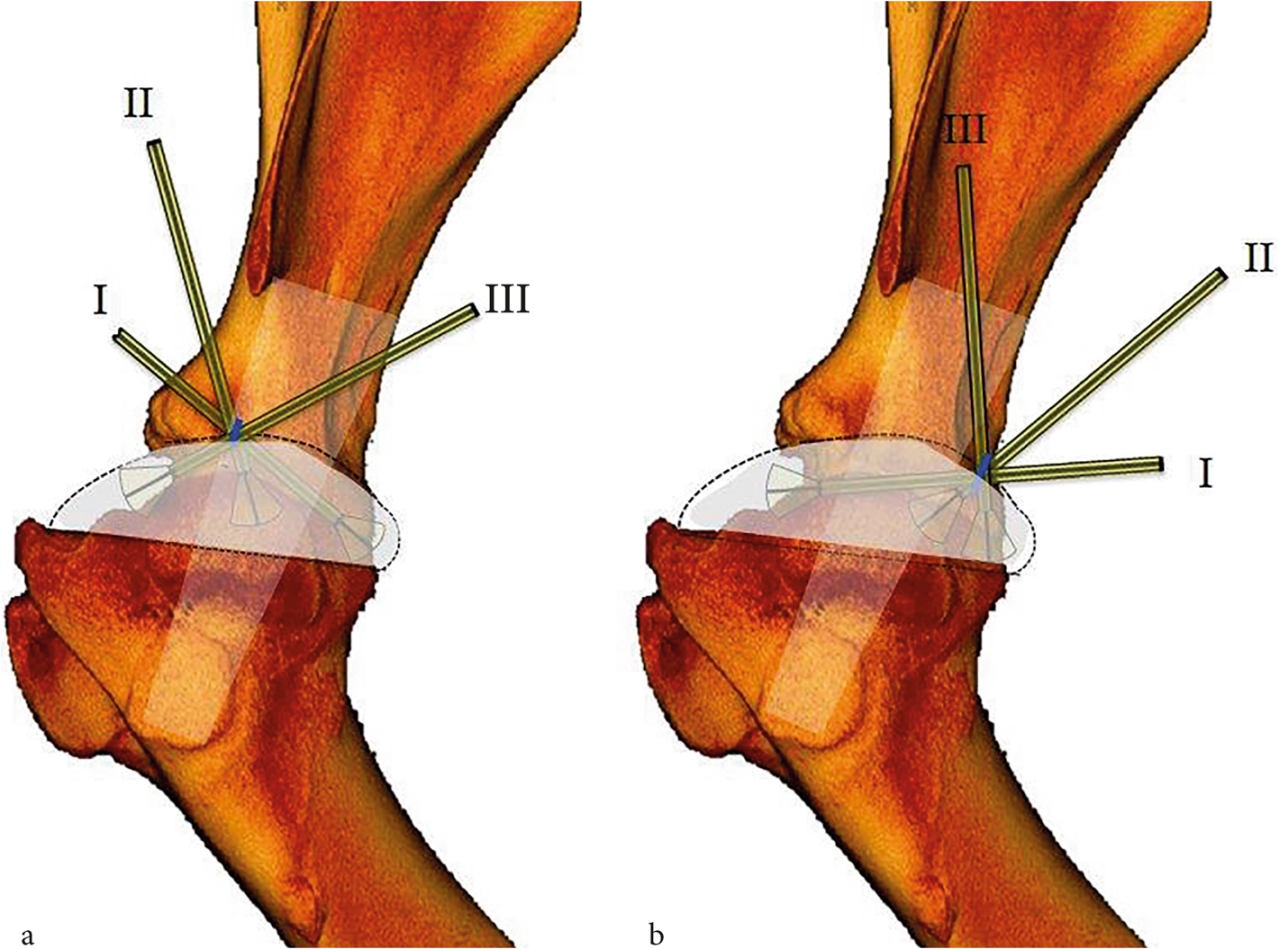
Computer tomographic recording of the left shoulder joint of a 5.2 years old cow, lateral view. Dotted lines mark schematically the delineations of the joint capsule, and the blue lines represent the incision lines immediately cranial to the tendon of the infraspinatus muscle (cranial approach; **a**) and 1 cm caudal to the tendon of the infraspinatus muscle (caudal approach; **b**). I, II & III represent the main directions of manipulation of the arthroscope within the joint

Afterwards, a systematic examination of the joint was performed. For the cranial approach, the investigation started with the tip of the arthroscope in the caudal aspect of the joint; afterwards, the arthroscope was slowly withdrawn along the lateral aspect of the joint. At the middle aspect of the glenoid, the joint was maximally distended by temporarily decreasing the outflow of water from the joint, and the arthroscope was carefully inserted below the glenoid and over the humeral head towards the medial side of the joint. The arthroscope was withdrawn to the lateral aspect and directed cranially, finally arriving in the cranial aspect of the joint (directions I, II and III, Fig. 4a).

As an alternative, the caudal approach was used. The arthroscope was oriented to the cranial aspect of the joint. After withdrawing the arthroscope caudally, the joint was maximally distended and the arthroscope carefully inserted below the glenoid and above the humeral head towards the medial aspect of the joint. Then, the arthroscope was withdrawn and moved caudally, and the caudolateral aspect of the joint was examined. Thereafter, the arthroscope was directed medially to examine the caudomedial part of the joint, and finally it was moved caudodistally to investigate the caudodistal cul-de-sac of the joint (directions I, II and III, Fig. 4b). For the first 20 limbs, the shoulder joints were examined with the limbs in horizontal and non-pulled position using the cranial and the caudal approaches. For the remaining 10 shoulders (age of 1 to 1451 days [median = 1 day), bodyweight of 40 to 684 kg (median = 50 kg)], after arthroscopy in horizontal non-pulled position, the arthroscopic investigation was repeated in abducted non-pulled position (25–30° abduction angle) and in horizontal manually pulled position for both approaches. The limb’s abduction was obtained by using a three-pod limb holder adjustable in height while pulling the limb was reached by putting continuous and constant traction on the limb with a rope attached on one side to the metacarpus III&/IV proximal to the fetlock joint an on the other side to a vertical rod fixed on the floor. Arthroscopic still pictures were taken of each step, and arthroscopic videos were continuously recorded for analysis later. The time of performing each procedure and the complications observed during the procedures were also recoded. The articular cartilage wear lines were scored according to the depth of the lesion. If lesions were restricted to the cartilage, they were scored as a partial-thickness lesions (the subchondral bone was not visible), but as deep lesions if they extended to the subchondral bone. The arthroscopic evaluations were performed within 4 h following euthanasia. The described arthroscopic procedures were always conducted by the same surgeon (first author).

### Histology

For the histological examination, a sample from a stranded band of unclear tissue composition that connected the cranial cul-de-sac with the cranial aspect of the humeral head was fixed in 4% paraformaldehyde and embedded in paraffin. Sections (2–5 μm) were routinely prepared, stained with hematoxylin-eosin (HE) and microscopically analyzed.

## Data Availability

The datasets used and/or analyzed during the current study are available from the corresponding author on reasonable request.

## Abbreviation

HE: Hematoxylin-eosin

## References

[1] Nickel R, Schummer A, Seiferle E. Eingeweide. lehrbuch der anatomie der Haustiere 2, vol. 2: Stuttgart: Georg Thieme Verlag; 2004. p. 67–72.

[2] Frandson RD, Wilke WL, Fails AD. Anatomy and physiology of farm animals: Hoboken: Wiley; 2009. p. 113–7.

[3] Altenbrunner-Martinek B, Grubelnik M, Kofler J (2007). Ultrasonographic examination of important aspects of the bovine shoulder-physiological findings. Vet J.

[4] Buergelt CD, Sisk D, Chenoweth PJ, Gamboa J, Nagus R (1996). Nutritional myodegeneration associated with dorsal scapular displacement in beef heifers. J Comp Pathol.

[5] Ferguson JG (1997). Surgical conditions of the proximal limb. Lameness in cattle.

[6] Nuss K (2003). Septic arthritis of the shoulder and hip joint in cattle: diagnosis and therapy. Schweiz Arch Tierheilkd.

[7] Nuss K, Ringer S, Meyer SW, Schade B, Ohlerth S (2007). Lameness caused by infection of the subtendinous bursa of the infraspinatus muscle in three cows. Vet Rec.

[8] Mearns R, Lewis H (2007). Flying scapula in cattle. Vet Rec.

[9] Altenbrunner-Martinek B, Starke A, Heppelmann M, Kofler J (2017). Disorders of the shoulder region in 21 cattle: clinical, ultrasonographic and radiographic findings. Berl Munch Tierarztl.

[10] Steiner A, Geissbuhler U, Siegrist A, Delley V, Mock L, Stoffel MH, Wegmuller M (2010). Electronic atlas of bovine radiology. Vet Radiol Ultrasound.

[11] Kofler J (1996). Arthrosonography--the use of diagnostic ultrasound in septic and traumatic arthritis in cattle--a retrospective study of 25 patients. Br Vet J.

[12] Hirsbrunner G, Steiner A (1998). Treatment of infectious arthritis of the radiocarpal joint of cattle with gentamicin-impregnated collagen sponges. Vet Rec.

[13] Kofler J, Geissbuhler U, Steiner A (2014). Diagnostic imaging in bovine orthopedics. Vet Clin North Am Food Anim Pract.

[14] Steiner A, Hirsbrunner G, Miserez R, Tschudi P (1999). Arthroscopic lavage and implantation of gentamicin-impregnated collagen sponges for treatment of chronic septic arthritis in cattle - 14 cases (1995-1997). Vet Comp Orthopaed.

[15] Kofler J, Martinek B (2005). New surgical approach to the plantar fetlock joint through the digital flexor tendon sheath wall and suspensory ligament apparatus in cases of concurrent septic synovitis in two cattle. Vet J.

[16] Starke A, Kehler W, Rehage J (2006). Arthrotomy and arthrodesis in the treatment of complicated arthritis of the fetlock joint in adult cattle. Vet Rec.

[17] Kofler J (2017). Pathogenesis and treatment of toe lesions in cattle including “nonhealing” toe lesions. Vet Clin North Am Food Anim Pract.

[18] Larde H, Nichols S (2014). Arthroscopy in cattle technique and Normal anatomy. Vet Clin N Am-Food A.

[19] Mulon PY, Desrochers A, Francoz D (2016). Surgical Management of Septic Arthritis. Vet Clin North Am Food Anim Pract.

[20] Trostle SS, Nicoll RG, Forrest LJ, Markel MD (1997). Clinical and radiographic findings, treatment, and outcome in cattle with osteochondrosis: 29 cases (1986-1996). J Am Vet Med Assoc.

[21] Hurtig MB (1985). Recent developments in the use of arthroscopy in cattle. Vet Clin North Am Food Anim Pract.

[22] Munroe GA, Cauvin ER (1994). The use of arthroscopy in the treatment of septic arthritis in two Highland calves. Br Vet J.

[23] Gaughan EM (1996). Arthroscopy in food animal practice. Vet Clin North Am Food Anim Pract.

[24] Blaser M, Bertagnoli A, Raber M, Nuss K, Rasekh M, Steiner A (2012). Arthroscopic approaches to the fetlock joint of adult cattle: a cadaver study. Vet J.

[25] Nichols S, Anderson DE (2014). Determination of the normal arthroscopic anatomy of the femoropatellar and cranial femorotibial joints of cattle. Can Vet J.

[26] Hagag U, Tawfiek MG, Brehm W (2015). Systematic arthroscopic investigation of the bovine stifle joint. Vet J.

[27] Larde H, Nichols S, Babkine M, Desrochers A (2016). Dorsal arthroscopic approach and intra-articular anatomy of the bovine Antebrachiocarpal and middle carpal joints. Vet Surg.

[28] Larde H, Nichols S, Babkine M, Desrochers A (2017). Arthroscopic approach and intra-articular anatomy of the dorsal and plantar synovial compartments of the bovine tarsocrural joint. Vet Surg.

[29] Hagag U, Tawfiek MG, Brehm W (2019). Palmar arthroscopic approach and intra-articular anatomy of the bovine carpal joints. Vet Surg.

[30] Bertone AL, McIlwraith CW (1987). Arthroscopic surgical approaches and intraarticular anatomy of the equine shoulder joint. Vet Surg.

[31] Nixon AJ (1987). Diagnostic and surgical arthroscopy of the equine shoulder joint. Vet Surg.

[32] Bertone AL, McIlwraith CW, Powers BE, Trotter GW, Stashak TS (1987). Arthroscopic surgery for the treatment of osteochondrosis in the equine shoulder joint. Vet Surg.

[33] Desrochers A, Francoz D (2014). Clinical Management of Septic Arthritis in cattle. Vet Clin N Am-Food A.

[34] Mansur DI, Manandhar P, Haque MK, Mehta DK, Duwal S, Timalsina B (2016). A study on variations of nutrient foramen of Humerus with its clinical implications. Kathmandu Univ Med J (KUMJ).

